# FruHis significantly increases the anti-benign prostatic hyperplasia effect of lycopene: A double-blinded randomized controlled clinical trial

**DOI:** 10.3389/fnut.2022.1011836

**Published:** 2022-11-03

**Authors:** Alireza Sadeghi, Ahmad Saedisomeolia, Leili Jalili-Baleh, Mehdi Khoobi, Mohammad Soleimani, Ali Mohammad Fakhr Yasseri, Mir Saeed Yekaninejad, Amirreza Farzin, Erfan Amini, Mohammad Reza Nowroozi

**Affiliations:** ^1^Department of Cellular and Molecular Nutrition, School of Nutritional Sciences and Dietetics, Tehran University of Medical Sciences (TUMS), Tehran, Iran; ^2^School of Human Nutrition, McGill University, Montreal, QC, Canada; ^3^Department of Medical Chemistry, Faculty of Pharmacy and Pharmaceutical Sciences, Tehran University of Medical Sciences, Tehran, Iran; ^4^Department of Radiopharmacy, Faculty of Pharmacy, Tehran University of Medical Sciences, Tehran, Iran; ^5^Department of Urology, Modares University Hospital, Shahid Beheshti University of Medical Sciences, Tehran, Iran; ^6^Endologist, Shariati Hospital, Alborz University of Medical Sciences, Karaj, Iran; ^7^Department of Epidemiology and Biostatistics, School of Public Health, Tehran University of Medical Sciences, Tehran, Iran; ^8^Uro-Oncology Research Center, Tehran University of Medical Sciences, Tehran, Iran

**Keywords:** lycopene, FruHis, tomato, benign prostatic hyperplasia, prostate specific antigen

## Abstract

**Background:**

For decades, lycopene was considered the main compound of tomato protecting benign prostatic hyperplasia (BPH). Recent animal studies suggest that a newly discovered compound “FruHis” boosts lycopene for its action. This study aimed to determine whether FruHis enhances the action of lycopene to modify the laboratory parameters and clinical outcomes of patients with BPH.

**Materials and methods:**

Current study was conducted on 52 BPH patients, who were randomly assigned into four groups of treatments: lycopene plus FruHis (*n* = 11, 25 mg/day lycopene and 10 mg/day FruHis), lycopene (*n* = 12, 25 mg/day lycopene), FruHis (*n* = 12, 10 mg/day FruHis), and placebo (*n* = 13). Patients received these supplements for 8 weeks.

**Results:**

FruHis intake strengthened the reducing effects of lycopene on insulin-like growth factor-1 (IGF-1) (−54.47 ± 28.36 ng/mL in the lycopene + FruHis group vs. −30.24 ± 46.69 ng/mL in the lycopene group), total prostate-specific antigen (TPSA) (−1.49 ± 4.78 ng/mL in the lycopene + FruHis group vs. −0.64 ± 2.02 ng/mL in the lycopene group), and symptom score (−4.45 ± 4.03 in the lycopene + FruHis group vs. −1.66 ± 5.41 in the lycopene group) in BPH patients. Such findings were also seen for body mass index (BMI) and waist circumference (WC). However, except for IGF-1, these reductions were not statistically significant compared with the placebo, and the intakes of lycopene and FruHis alone, however, were clinically important. Such effects of lycopene and FruHis were not seen for free PSA (FPSA) and FPSA/TPSA ratio.

**Conclusion:**

Despite the non-significant effects of lycopene and FruHis, it seems that FruHis intake strengthens the beneficial effects of lycopene on IGF-1, TPSA, and symptom scores among BPH patients.

**Clinical trial registration:**

[www.irct.ir], identifier [IRCT20190522043669N1].

## Introduction

Benign prostatic hyperplasia (BPH) is prevalent among 50% of men with the age range of ≥50 years and 90% of ≥80-year-old men (1, 2). This disorder is characterized by a non-malignant enlargement of the prostate gland that is due to the proliferation of epithelial and stromal cells in this gland (2, 3). BPH is associated with an increased smooth muscle tone and obstruction of the proximal urethra leading to increased urinary frequency, nocturia, urinary incontinence, and voiding (slow and/or weak stream); all of these symptoms decrease the quality of life of patients considerably (3, 4).

Different types of pharmaceutical therapies such as alpha-blockers and 5-alpha-reductase inhibitors have been suggested to control BPH outcomes (5, 6). However, these medications may cause side events including erectile dysfunction, decreased libido, dizziness, and hypotension (5). Hence, “complementary” or “alternative therapies” with limited adverse events are taken into consideration long ago. Among these approaches, much attention has been paid to lycopene, a lipid-soluble antioxidant compound, which is one of the main dietary carotenoids mostly found in tomato and its products (7, 8). It has been reported that lycopene is the strongest antioxidant among carotenoids. It has been shown that lycopene has a role in the prevention and management of BPH mainly through its antioxidant activity, inhibition of cell cycle progression, induction of apoptosis, increasing of gap-junctional cell communication, and inhibition of insulin-like growth factor I signal transduction (9–11).

Recent studies have shown that other constituents of cooked tomatoes may contribute to BPH prevention (12). Also, tomato processing, particularly heat, may alter tomato constituents and thereby changes the BPH-protective effects of tomato (13). During heat-processing, the tomato loses the activity of its known natural antioxidants, such as ascorbate, whereas the total antioxidant activity of the heat-processed tomato does not change and may often increase (13–15). In addition, observational studies reported that intake of heat-processed tomato products, compared to raw tomato, was associated with a lower risk of BPH and prostate cancer (PC) (16). However, it is still unclear which constituents are produced during the thermal process of tomato and which reaction is involved in this regard.

One of the most common chemical reactions during heat-processing is the Maillard reaction, which is a process in which an amino acid attaches a reducing sugar. This process is responsible for the browning and specific flavoring of baked, roasted, and dried foods, such as toasted bread and fried potatoes (17). This reaction produces ketosamines, such as FruHis (attachment of Fructose to Histidine), that are indigestible and partially absorbed into the bloodstream (18). An experimental study on rats has shown that FruHis exerts antioxidant and anti-cancer properties (12). However, it is not clear whether FruHis provides higher PC-protective effects for heat-processed tomatoes, compared to raw tomatoes. The interaction between lycopene and FruHis against prostate tumorigenesis is another question. Until now, no study has answered these questions. Therefore, the current study was conducted to determine the effect of lycopene and FruHis supplementation, combined and alone, on laboratory parameters and clinical outcomes of men with BPH.

## Materials and methods

### Participants

This study was a double-blind randomized controlled clinical trial that was conducted by the Department of Cellular and Molecular Nutrition, Tehran University of Medical Sciences, Tehran, Iran, from January 2020 to November 2021. Outpatients with BPH were recruited from educational and therapeutic centers of Tehran University of Medical Sciences. BPH was diagnosed by an expert urologist according to patient history, digital rectal examination (DRE), and laboratory findings, including serum prostate-specific antigen (PSA). According to the sample size formula suggested for intervention studies, considering a type I error of 5% (α = 0.05), type II error of 20% (β = 0.20, power = 80%), and serum PSA levels as the most conservative variable, we required sample size of 12 individuals for each group. However, 13 patients were enrolled in the intervention groups to make sure that the study powers significantly after the probable dropouts of the patients.

### Inclusion and exclusion criteria

**FIGURE 1:**
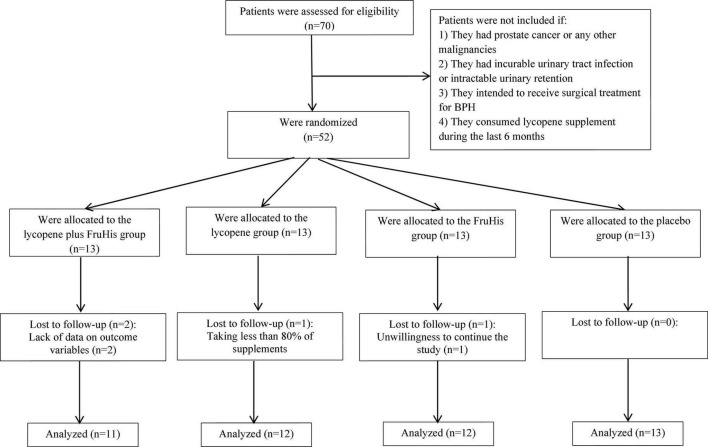
The flowchart of the current study.

Patients were considered eligible for study entry if they had an age range between 50 and 70 years and had confirmed BPH based on clinical diagnosis by an expert urologist using clinical examination, DRE, and paraclinical tests including serum PSA. Criteria of BPH diagnosis included a negative test of the digital rectal exam (DRE) and PSA levels between 4 and 10 ng/mL. To rule out prostatic cancer among suspicious cases, we assessed findings from the prostate biopsy and excluded patients with PSA levels of >10 ng/mL. Patients were not included if they (1) had PC or any other malignancies; (2) had incurable urinary tract infection or intractable urinary retention; (3) intended to receive surgical treatment for BPH; (4) consumed supplements containing lycopene during the past 6 months, and (5) had a history of allergy to tomato or its products. Moreover, we excluded individuals who changed the type or dosage of their medications (related to BPH) during the intervention, patients who consumed unusual amounts of tomato or its products during the study, those that were not willing to continue with the intervention process, patients who suffered from probable complications related to prescribed supplements, and individuals who consumed less than 80% of lycopene, FruHis, or placebo supplements during the trial. The flowchart of this study is shown in Figure 1.

### Ethics

After the selection of patients, they were asked to complete a written informed consent to participate in the current study. The Ethics Committee of Tehran University of Medical Sciences has approved the study protocol of this clinical trial (IR.TUMS.VCR.REC.1397.951). Moreover, this clinical trial has been registered in the Iranian Registry of Clinical Trials^1^ with the reference number IRCT20190522043669N1.

### Study design and intervention

After selecting participants based on the mentioned criteria, first, they were stratified based on age groups (55–60 and 60–75 years) and BMI (18.5–24.9 and 25–30 years), and then, were randomly allocated to the four intervention groups, including lycopene plus FruHis, lycopene, FruHis, and placebo groups. In this way, we identified patients with the same condition in terms of age and BMI. Then, to conduct random allocation, an identification code was given to each participant, the codes of participants with the same age and BMI were stated in a lottery container, and finally, they were randomly assigned to the four intervention groups. Random allocation was performed by an independent person who was unaware of the aim of our study. Patients in the lycopene plus FruHis group received two capsules per day; one contained 25 mg/day of lycopene and one contained 10 mg/day of FruHis. Patients in the lycopene group received one capsule containing 25 mg/day of lycopene and one placebo capsule per day. Patients in the FruHis group received one capsule containing 10 mg/day of FruHis and one placebo capsule every day. In the placebo group, patients received two placebo capsules per day. Placebo capsules contained starch. At the baseline study, all capsules that should be consumed during the study were given to patients. The length of the intervention was 8 weeks because previous studies have shown that lycopene supplementation during 4–8 weeks with a dosage between 15 and 30 mg/day reduced BPH outcomes such as serum levels of PSA (19). However, there is no evidence of the best dosage of lycopene. In the current study, we aimed to answer the question: Does FruHis increase the beneficial effect of lycopene with the same dosage and intervention duration compared with the previous studies? Since we found no study on FruHis supplementation and given that the FruHis dosage in cooked tomato is less than lycopene (18), we administered 10 mg/day FruHis in the current trial.

### Compliance to study protocol

To increase the compliance of participants, phone messages were sent to participants to remind the consumption of prescribed supplements on weekly base time points. In addition to the message, taking supplements was monitored by phone calls every other week. Participants were asked to return the empty capsule packs at end of the trial, to make sure that the boxes are empty. Participant’s compliance was assessed using the following formula: (number of used capsules/all given capsules) × 100. Acceptable compliance with the study was considered as 80% or over. We assessed primary and secondary outcome variables at the study baseline and end of the trial.

To assess dietary intakes during the study, participants were asked to fill a 3-day dietary record (Supplementary file, section A) in the first 2 weeks and a 3-day dietary record in the last 2 weeks. The 3-day records included 2 weekdays and a weekend. Participants were asked to fulfill the dietary records based on household measures and then, the household measures were converted to grams using available booklets for domestic foods. The mean dietary intakes in the six dietary records were considered as usual dietary intakes during the study. Moreover, the dietary intakes (in grams) were converted to nutrient intakes using the US National Nutrient Databank modified for Iranian Foods (20, 21). In addition to dietary intake, the physical activity throughout the intervention period was assessed two times using 1-day physical activity records; one in the first 2 weeks and one in the last 2 weeks of the intervention (Supplementary file, section B). All the participants were trained to fill out their physical activity records. Participants were asked not to change their physical activity and dietary intake during the study.

### Preparation of supplements

Lycopene and placebo supplements were provided by the Pourateb Pharmaceutical Company, Tehran, Iran. To prepare FruHis, the method developed by Mossine et al. (18, 22) was used. Briefly, FruHis is synthesized from an aqueous solution of food-grade L-histidine, glacial acetic acid, and D-glucose. Then, the solution was purified to provide non-hygroscopic crystalline powder, which was free of any detectable impurities. The melting point was determined on a Kofler hot stage apparatus. The IR spectra (KBr disks) were recorded by the Nicolet FT-IR Magna 550 spectrometer. ^1^H and ^13^C NMR spectra were recorded using the Varian-INOVA 500 MHz instrument. Mass analysis of the compound was determined with the Agilent Technology (HP), Electron Impact 70 eV. Nα-(1-Deoxy-D-fructos-1-yl)-L-histidine. Mp: 130–140 (dec.); IR (KBr, cm^–1^) ν_*max*_: 3,300 (NH), 2,930 (C-H), 1,621 (*C* = O); ^1^H NMR (D_2_O, 500 MHz) δ: 8.53 (s, 1H, Imidazolyl), 7.27 (s, 1H, Imidazolyl), 4.07–3.32 (m, β-pyranose and α-β-furanose forms). ^13^C NMR (D_2_O, 125 MHz) δ: 171.1, 133.8, 126.6, 117.8, 95.1, 73.9, 69.7, 69.1, 68.7, 63.8, 61.6, 24.3.

The appearance of the FruHis capsules, such as color, shape, size, and packaging, was identical to the lycopene and placebo capsules.

### Assessment of variables

A researcher-made questionnaire was used to collect data on age, education, marital status, ethnicity, economic status, smoking, medical history, and nutritional supplement use at the study baseline. At the baseline and end of the trial, the primary outcome variables including serum levels of total PSA (TPSA), free PSA (FPSA), FPSA/TPSA ratio, insulin-like growth factor-1 (IGF-1), disease severity, quality of life (QoL), and secondary outcome variables including body mass index (BMI) and waist circumference (WC) were measured.

### Biochemical assessments

After 8 h of fasting, 5 mL venous blood samples were collected from each participant at the beginning and end of the trial. After sampling, serum was extracted from the blood sample and then, was stored at –70°C until further analysis. TPSA and FPSA were measured using the commercial kits of chemiluminescence enzyme immunoassay (CLEIA) (Roche, Germany). Also, serum concentrations of IGF-1 were measured using the commercial kits of enzyme-linked immunosorbent assay (ELISA) (Mediagnost, Germany).

### Assessment of symptoms and quality of life

American Urological Association (AUA) symptom index was used to assess the obstructive and irritative voiding symptoms (23). The AUA symptom index contains seven items evaluating BPH symptoms including incomplete emptying, frequency, intermittency, urgency, weak stream, hesitancy, and nocturia. Each item can be scored from 0 to 5. By summing up the scores, a score between 1 and 35 is achieved. In this scoring outcome measure, greater scores show higher severity of BPH symptoms. The total score of BPH symptoms was considered as an outcome variable in the current study. In addition to the seven items mentioned above, the updated version of the AUA symptom index contains one disease-specific QoL question as follows (23): If you were to spend the rest of your life with your urinary condition just the way it is now, how would you feel about that? The response categories for this question were “delighted” (score 0), “pleased” (score 1), “mostly satisfied” (score 2), “mixed” (score 3), “mostly dissatisfied” (score 4), “unhappy” (score 5), and “terrible” (score 6). In the current study, we considered the score of QoL improvement as an outcome variable. QoL improvement was considered as changing the scores from 4–6 to 0–3 throughout the trial.

The World Health Organization (WHO) confirmed the reliability and validity of the updated version of the AUA symptom index and considered it the International Prostate Symptom Score (IPSS) (24). The IPSS was translated to Persian and the validity and reliability of this version were confirmed in Panahi et al. study (25).

### Anthropometric measurements

All anthropometric measures were assessed according to the US National Institutes of Health protocols (26). Weight was measured using a digital scale at the state of minimum clothing without shoes to the nearest 100 g. Height was measured using a standard stadiometer, without shoes, to the nearest 0.5 cm. BMI was calculated as weight (kg)/height (m^2^). WC was measured using a strip meter at mid-distance intervals between the super elliptic bone and the last gear, to the nearest 0.5 cm.

### Statistical analysis

The normality of the distribution of outcome variables was assessed using the Kolmogorov–Smirnov test. Among baseline and dietary data, three variables including age and dietary intakes of vitamin D and selenium were not normal-distributed and therefore we used non-parametric tests for these variables. Among outcome variables, the distribution of TPSA and BMI was not normal. We normalized these variables using the log transformation to avoid using non-parametric tests for outcome variables. To examine differences in continuous baseline and dietary variables across the four intervention groups, one-way analysis of variance (ANOVA) (for normal-distributed variables) and Kruskal–Wallis (for non-normally distributed variables) were employed. In addition, the Chi-square test is used to assess the distribution of categorical variables across the four intervention groups. To assess the effect of interventions on outcome variables in each group, a paired-sample *t*-test was used. Moreover, to compare changes in outcome variables across the four intervention groups, the ANOVA test was employed. The two-by-two comparison was done using the Bonferroni test. In addition, multivariate analysis of covariance (ANCOVA), as a general linear model, was used to examine the effects of lycopene and FruHis supplementation on outcome variables. In this analysis, we controlled for baseline values of outcome variables to detect an independent effect. All statistical analyses were conducted using the SPSS software version 18 (SPSS, Inc. Chicago, IL, USA). *P* < 0.05 will be considered significant.

## Results

### Characteristics of the participants

From all recruited patients (*n* = 52), four were excluded due to taking less than 80% of supplements (*n* = 1), unwillingness to continue the study (*n* = 1), and lack of data on outcome variables (*n* = 2). In total, 48 patients with complete data were included in the statistical analysis: 11 patients in the lycopene plus FruHis group, 12 patients in the lycopene group, 12 patients in the FruHis group, and 13 patients in the placebo group. Regarding adherence to interventions, it is found that all patients included in the analysis consumed 100% of the capsules throughout the study period.

The baseline characteristics of participants in the four intervention groups are shown in Table 1. We found no significant difference between the four intervention groups in terms of demographic variables, smoking habits, anthropometric measures, disease history, and supplement consumption history. In addition, the daily dietary intakes of participants throughout the trial, particularly tomato and its products, were not different across the four intervention groups (Table 2).

**TABLE 1:** Baseline characteristics of participants in the intervention groups.

Variables	Lycopene plus FruHis group (*n* = 11)	Lycopene group (*n* = 12)	FruHis group (*n* = 12)	Placebo group (*n* = 13)	*P*-value*
Age (year)	65.00 ± 9.64	62.16 ± 6.17	64.61 ± 9.13	65.15 ± 7.17	0.78
Weight (kg)	77.48 ± 14.57	77.90 ± 21.56	74.56 ± 11.73	80.14 ± 15.00	0.86
BMI (kg/m^2^)	25.99 ± 3.52	25.98 ± 4.66	25.64 ± 3.78	26.27 ± 4.80	0.98
WC (cm)	97.72 ± 9.00	98.25 ± 12.85	100.25 ± 8.85	101.81 ± 9.47	0.77
University educated (%)	54.5	33.3	41.7	53.8	0.68
Fars ethnicity (%)	72.7	75.0	75.0	84.6	0.89
Economic status (weak) (%)	27.3	58.3	58.3	23.1	0.13
Current smoker (%)	9.1	41.7	0	30.8	0.05
Married (%)	100	91.7	91.7	76.9	0.30
PA (Met-h/wk)	2277.7 ± 423.3	2264.4 ± 605.5	2189.5 ± 310.7	2392.4 ± 442.3	0.78
Supplement use (%)	54.5	50.0	50.0	46.2	0.98
**Disease history**					
CVD (%)	45.5	33.3	25.0	30.8	0.76
Hypertension (%)	9.1	25.0	16.7	7.7	0.60
Diabetes (%)	45.5	33.3	16.7	15.4	0.30
Thyroid diseases (%)	9.1	8.3	25.0	15.4	0.63

Data are presented as mean (± SD) or percent. BMI, body mass index; WC, waist circumference; CVD, cardiovascular disease; PA, physical activity.

*Obtained from one-way analysis of variance (ANOVA) (for normal-distributed continuous variables) and Kruskal–Wallis (for non-normally distributed continuous variables) or Chi-square test (for categorical variables).

**TABLE 2:** Dietary intakes of participants throughout the trial in the intervention groups.

Variables	Lycopene plus FruHis group (*n* = 11)	Lycopene group (*n* = 12)	FruHis group (*n* = 12)	Placebo group (*n* = 13)	*P*-value*
Energy (Kcal)	1473 ± 275	1313 ± 329	1443 ± 234	1392 ± 476	0.71
Protein (g/day)	52.75 ± 11.31	48.08 ± 11.35	52.96 ± 11.69	52.41 ± 17.42	0.78
Carbohydrate (g/day)	189.10 ± 45.10	164.43 ± 45.67	177.32 ± 30.90	175.20 ± 61.27	0.66
Fat (g/day)	58.45 ± 14.68	53.15 ± 14.73	60.13 ± 10.62	55.23 ± 20.45	0.69
SFA (g/day)	29.46 ± 6.86	28.72 ± 8.85	26.85 ± 8.49	28.01 ± 8.60	0.89
PUFA (g/day)	42.37 ± 10.93	39.73 ± 10.11	39.64 ± 15.42	39.75 ± 9.87	0.93
Fiber (g/day)	10.83 ± 2.81	10.00 ± 3.02	10.67 ± 3.48	10.19 ± 3.22	0.90
Vitamin C (mg/day)	113.34 ± 44.82	95.54 ± 63.99	130.32 ± 62.23	133.01 ± 76.01	0.44
Vitamin E (mg/day)	3.96 ± 2.52	3.80 ± 1.41	3.66 ± 1.30	4.42 ± 2.10	0.76
Vitamin D (mg/day)	0.58 ± 1.19	0.45 ± 0.96	0.11 ± 0.18	0.51 ± 1.00	0.31
Iron (mg/day)	21.09 ± 5.59	18.56 ± 5.73	17.51 ± 6.43	19.85 ± 6.33	0.52
Selenium (mg/day)	0.03 ± 0.04	0.02 ± 0.01	0.03 ± 0.02	0.02 ± 0.03	0.39
Tomato and its products (g/day)	20.06 ± 15.24	14.70 ± 14.05	21.21 ± 17.55	14.67 ± 11.99	0.56

Data are presented as mean (± SD). SFA, saturated fatty acid; PUFA, polyunsaturated fatty acid.

*Obtained from one-way analysis of variance (ANOVA) (for normal-distributed variables) and Kruskal–Wallis (for non-normally distributed variables).

### Intervention

**FIGURE 2:**
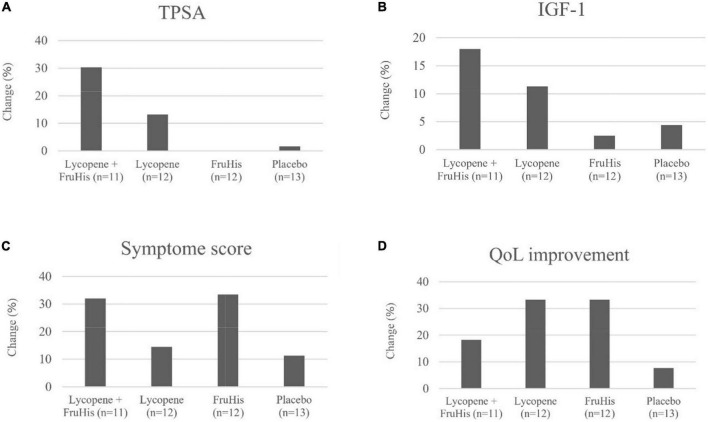
The effects of lycopene and FruHis supplementation, combined and alone, on TPSA **(A)**, IGF-1 **(B)**, symptom score **(C)**, and QoL improvement **(D)** among patients with BPH. QoL improvement was considered as changing the scores of 4–6 to 0–3 (based on QoL question in IPSS) throughout the trial. IPSS, international prostate symptom score; QoL, quality of life; TPSA, total prostate-specific antigen; IGF, insulin-like growth factor; BPH, benign prostatic hyperplasia.

The influences of lycopene and FruHis supplementation on primary and secondary outcome variables are indicated in Table 3. Our results showed that placebo and FruHis alone did not induce any change in TPSA, while lycopene induced a 13.2% reduction (-0.64 ± 2.02 ng/mL) in TPSA. However, when patients consumed a combination of FruHis and lycopene, FruHis increased the effect of lycopene from 13 to 30.3% (-1.49 ± 4.78 ng/mL) (Table 3 and Figure 2). This reduction was not significant compared with the placebo group and the intake of lycopene and FruHis alone. Also, we observed the same pattern for IGF-1, symptom score, BMI, and WC, when FruHis improved the effect of lycopene on IGF-1 from 11.3 to 18% (-54.47 ± 28.36 ng/mL in the lycopene + FruHis group vs. –30.24 ± 46.69 ng/mL in the lycopene group), on symptom score from 14.5 to 32% (-4.45 ± 4.03 in the lycopene + FruHis group vs. –1.66 ± 5.41 in the lycopene group), on BMI from 0.38 to 2.1% (-0.55 ± 0.90 kg/m^2^ in the lycopene + FruHis group vs. –0.10 ± 0.30 kg/m^2^ in the lycopene group), and on WC from 0.2 to 0.7% (-0.68 ± 0.46 cm in the lycopene + FruHis group vs. –0.20 ± 0.86 cm in the lycopene group) (Figure 2 and Table 3). Comparing the end of the trial with baseline values, the observed changes in IGF-1, symptom score, and WC were statistically significant in the lycopene plus FruHis group (Table 3). However, between-group comparisons led to non-significant results. For FPSA, no significant change was seen in all intervention groups. In terms of FPSA/TPSA ratio, when comparing baseline and end-of-trial values, the effect of lycopene supplementation alone was more than the combination of lycopene and FruHis (5.44 ± 7.59% in the lycopene plus FruHis group vs. 2.77 ± 10.24% in the lycopene group); however, this difference was not statistically significant (Table 3). When the analyses were adjusted for baseline values of outcome variables, the reducing effect of lycopene plus FruHis supplementation on IGF-1 remained significant (Table 4).

**TABLE 3:** Means of outcome variables at baseline and end of the trial and changes during the trial in the intervention groups.

	Week 0	Week 8	Mean change	Change %	*P* *	*P* **
IGF-1 (ng/mL)						0.12
Lycopene plus FruHis group (*n* = 11)	302.66 ± 87.24	248.18 ± 73.17	−54.47 ± 28.36	18.0	<0.001	
Lycopene group (*n* = 12)	265.56 ± 51.29	235.31 ± 54.53	−30.24 ± 46.69	11.3	0.04	
FruHis group (*n* = 12)	318.04 ± 63.63	309.98 ± 55.17	−8.06 ± 38.00	2.5	0.47	
Placebo group (*n* = 13)	277.72 ± 85.63	265.35 ± 43.85	−12.37 ± 73.05	4.4	0.55	
FPSA (ng/mL)						0.47
Lycopene plus FruHis group (*n* = 11)	0.73 ± 0.48	0.70 ± 0.43	−0.02 ± 0.35	2.7	0.84	
Lycopene group (*n* = 12)	0.71 ± 0.19	0.91 ± 0.51	0.20 ± 0.57	28.1	0.24	
FruHis group (*n* = 12)	0.84 ± 0.71	0.93 ± 0.45	0.10 ± 0.35	11.9	0.34	
Placebo group (*n* = 13)	0.59 ± 0.32	0.59 ± 0.33	−0.004 ± 0.19	0.67	0.93	
TPSA (ng/mL)						0.51
Lycopene plus FruHis group (*n* = 11)	4.91 ± 5.27	3.42 ± 1.94	−1.49 ± 4.78	30.3	0.32	
Lycopene group (*n* = 12)	4.82 ± 1.68	4.18 ± 1.39	−0.64 ± 2.02	13.2	0.29	
FruHis group (*n* = 12)	4.77 ± 3.67	4.76 ± 2.69	−0.002 ± 1.59	0	0.99	
Placebo group (*n* = 13)	4.27 ± 2.82	4.19 ± 3.01	−0.07 ± 1.00	1.6	0.78	
FPSA/TPSA (%)						0.72
Lycopene plus FruHis group (*n* = 11)	18.76 ± 10.30	21.53 ± 5.82	2.77 ± 10.24	14.7	0.39	
Lycopene group (*n* = 12)	15.56 ± 3.99	21.00 ± 7.20	5.44 ± 7.59	35.0	0.03	
FruHis group (*n* = 12)	18.59 ± 7.85	21.24 ± 7.28	2.64 ± 4.06	14.2	0.04	
Placebo group (*n* = 13)	15.58 ± 6.67	17.69 ± 11.36	2.11 ± 8.20	13.5	0.37	
Symptom score^a^						0.12
Lycopene plus FruHis group (*n* = 11)	13.90 ± 7.73	9.45 ± 7.72	−4.45 ± 4.03	32.0	0.004	
Lycopene group (*n* = 12)	11.41 ± 5.08	9.75 ± 6.28	−1.66 ± 5.41	14.5	0.30	
FruHis group (*n* = 12)	18.25 ± 8.82	12.12 ± 8.64	−6.12 ± 7.27	33.5	0.01	
Placebo group (*n* = 13)	11.07 ± 7.91	9.81 ± 7.15	−1.26 ± 5.39	11.3	0.41	
BMI (kg/m^2^)						0.17
Lycopene plus FruHis group (*n* = 11)	25.99 ± 3.52	25.43 ± 3.29	−0.55 ± 0.90	2.1	0.06	
Lycopene group (*n* = 12)	25.98 ± 4.66	25.87 ± 4.70	−0.10 ± 0.30	0.38	0.24	
FruHis group (*n* = 12)	25.95 ± 3.83	25.86 ± 3.90	−0.08 ± 0.20	0.30	0.23	
Placebo group (*n* = 13)	27.12 ± 4.69	26.91 ± 4.69	−0.21 ± 0.50	0.77	0.19	
WC (cm)						0.38
Lycopene plus FruHis group (*n* = 11)	98.40 ± 9.29	97.72 ± 9.00	−0.68 ± 0.46	0.69	0.001	
Lycopene group (*n* = 12)	98.45 ± 12.61	98.25 ± 12.85	−0.20 ± 0.86	0.20	0.42	
FruHis group (*n* = 12)	100.40 ± 9.04	100.25 ± 8.85	−0.15 ± 0.41	0.15	0.27	
Placebo group (*n* = 13)	102.27 ± 9.42	101.81 ± 9.47	−0.45 ± 1.12	0.44	0.21	

Data are presented as mean (± SD). IGF, insulin-like growth factor; FPSA, free prostate-specific antigen; TPSA, total prostate-specific antigen; IPSS, international prostate symptom score; BMI, body mass index; WC, waist circumference.

^a^Obtained from IPSS.

*Obtained from the paired sample *t*-test.

**Obtained from the one-way analysis of variance (ANOVA). The two-by-two comparison was done using the Bonferroni test.

**TABLE 4:** Adjusted mean changes of outcome variables throughout the trial in the intervention groups.

Variables	Lycopene plus FruHis group (*n* = 11)	Lycopene group (*n* = 12)	FruHis group (*n* = 12)	Placebo group (*n* = 13)	*P* *
IGF-1	−49.17 ± 11.94^a^	−41.09 ± 11.57	3.92 ± 11.61	−17.92 ± 10.99	0.01
FPSA	−0.016 ± 0.10	0.20 ± 0.09	0.15 ± 0.10	−0.05 ± 0.09	0.20
TPSA	−1.36 ± 0.54	−0.56 ± 0.52	0.04 ± 0.52	−0.30 ± 0.50	0.42
FPSA/TPSA (%)	3.49 ± 2.18	4.81 ± 2.09	3.29 ± 2.09	1.49 ± 2.01	0.71
Symptom score^a^	−4.36 ± 1.59	−2.30 ± 1.54	−4.78 ± 1.60	−1.99 ± 1.49	0.51
BMI	−0.56 ± 0.16	−0.11 ± 0.16	−0.08 ± 0.17	−0.20 ± 0.16	0.17
WC	−0.68 ± 0.24	−0.21 ± 0.23	−0.14 ± 0.25	−0.44 ± 0.24	0.38

Data are presented as mean (± SE) adjusted for baseline values of outcome variables. IGF, insulin-like growth factor; FPSA, free prostate-specific antigen; TPSA, total prostate-specific antigen; IPSS, international prostate symptom score; BMI, body mass index; WC, waist circumference.

^a^Obtained from IPSS and significant compared with the FruHis group.

*Obtained from one-way analysis of covariance (ANCOVA). The two-by-two comparison was done using the Bonferroni test.

In terms of QoL, an improvement was seen in the lycopene plus FruHis (18.2%), lycopene (33.3%), and FruHis (33.3%) groups without any remarkable changes in the placebo group (7.7%) (Figure 2).

## Discussion

Based on the current literature review, this was the first study examining the effects of lycopene and FruHis supplementation, combined and alone, on laboratory parameters, disease symptoms, and QoL of patients with BPH. We found that intake of FruHis increases the beneficial effects of lycopene on TPSA, IGF-1, symptom score, BMI, and WC in patients with BPH. However, this increase for mentioned variables, except for IGF-1, was statistically non-significant compared with the placebo and intakes of lycopene and FruHis alone.

Benign prostatic hyperplasia is one of the most common diseases among men that is associated with chronic and progressive lower urinary tract symptoms (LUTS) or chronic complications, resulting in many health complications for men (27–29). In recent decades, many researchers have shown the beneficial effects of tomato or lycopene consumption on clinical outcomes of BPH patients (30). However, there is evidence indicating other constituents of tomato, particularly heat-processed tomato products, may have a role in the beneficial effect (12). Obviously, due to the heating process, there will be an increased bioaccessibility and bioavailability of lycopene and other lipid-soluble compounds compared to raw tomatoes. FruHis is a ketosamine that is produced during the heat processing of tomato products by attaching histidine to fructose (18). However, according to the previous studies, it is not clear if FruHis is responsible for the anti-BPH effects of tomatoes. In the current study, we found that when FruHis was combined with lycopene, it induces a non-significant decrease in TPSA from 13.2 to 30.3%. This effect is in the line with previous findings on heat-processed tomato products containing a high amount of FruHis and lycopene. Paur et al. reported that 3 weeks of interventions with tomato products reduces serum concentrations of PSA in patients with BPH (31). Moreover, in a review article, Basu et al. concluded that consumption of processed tomato products has a higher PSA-reducing effect compared to the intake of lycopene supplements alone (32). It should be noticed that there are several studies indicating a beneficial effect of lycopene supplementation alone on PSA levels. In a randomized clinical trial, Schwarz et al. reported that 24 weeks of lycopene supplementation (15 mg/day) significantly reduces TPSA in men with BPH (33). The same significant reduction was reported by another study on men with high-grade prostatic intraepithelial neoplasia (HGPIN) after an intervention with 8 mg/day of lycopene for 48 weeks (34). In the current study, it must be noticed that the reducing effect of lycopene + FruHis supplementation on TPSA was not statistically significant compared to the placebo group. This might be explained by the low sample size of the current pilot study. In addition, the low dosage of lycopene and FruHis might be another reason. As seen in Table 1, the mean BMI of study participants was >25 kg/m^2^, and therefore, 25 mg/day of lycopene and 10 mg/day FruHis might be inadequate for patients with overweight. In line with this claim, Cumar et al. assessed the effects of supplementation with 30 and 45 mg/day of lycopene on BPH patients with overweight and reported a non-significant effect compared with a placebo group (35). In addition, age is another factor affecting the efficacy of lycopene. Older patients may have a lower capacity for lycopene digestion and absorption, and therefore, need higher amounts of lycopene compared with younger patients.

In the current study, we found that FruHis supplementation increases the reducing effect of lycopene on IGF-1 concentrations from 11.3 to 18%. In the same line with our findings, Riso et al. reported a significant reducing effect on IGF-1 levels in healthy subjects due to the consumption of tomato drinks with a high amount of lycopene and probable content of FruHis (36). However, this significant effect was not seen in the study by Gann et al. who investigated the influence of lycopene-rich tomato extract on IGF-1 (37). The lack of significant effect in the Gann et al. study might be explained by a probable low amount of FruHis in the tomato extract administered. Overall, it seems that the combined intake of lycopene and FruHis, similar to the intake of tomato products containing both lycopene and FruHis, has a better reducing effect on IGF-1 compared to their intakes alone. In the current study, the baseline values of IGF-1 in the lycopene plus FruHis group were higher than the values in the lycopene group. This might be a reason for the greater reduction of IGF-1 in the combination group compared with the lycopene alone. However, when we adjusted the analyses for the baseline values of IGF-1, the significant effect of lycopene plus FruHis intake on IGF-1 remained significant.

The exact mechanisms through which lycopene and FruHis affect serum IGF-1 and TPSA levels are unknown. Although the physiological effects of FruHis have not been studied yet, there are some studies on lycopene (38, 39). It is proposed that lycopene contributes to some physiological pathways through inhibition of cell cycle progression, interleukin-6 expression, and androgen activation and signaling (38, 39). Hence, lycopene interferes with estrogen and androgen signaling, which has been proven to influence the production of IGF-1 and TPSA (40, 41). Also, oxidative stress contributes to the pathophysiology of BPH (42). Therefore, the antioxidant properties of lycopene may have another mechanism involved in the protective effects of lycopene on BPH. In the current study, FruHis intake strengthened the beneficial effects of lycopene. In an experimental study on rat prostate tumorigenesis, it had been shown that FruHis exerts antioxidant and anti-cancer properties (12). Since both lycopene and FruHis have antioxidant and anti-cancer effects, FruHis intake may have a synergistic effect on the beneficial effects of lycopene.

We also found that a combination of lycopene and FruHis intake, compared to lycopene intake alone, had a better reducing effect on symptom scores in BPH men. In addition, we found an improvement in QoL in the lycopene plus FruHis group compared to the placebo group. However, these positive effects were not statistically significant, and the size of changes is clinically notable. In agreement with our findings, Cormio et al. reported that consumption of whole tomato food supplement (WTFS), which may contain both lycopene and FruHis, decreases the symptom score from 9.05 to 7.15 and improves QoL, about one score, in patients with BPH (43). In the mentioned study, these changes were not significant compared to the control group. Both our study and Cormio et al. study had a low sample size and short duration of intervention that which may explain the non-significant positive effects of lycopene plus FruHis compared to the control group.

It must be kept in mind that our findings might be affected, to some extent, by the baseline characteristics of participants. For instance, patients in the lycopene group were more likely to be smokers and have hypertension compared with the lycopene plus FruHis group. This may explain the lower reduction of IGF-1 in the lycopene group compared with the lycopene plus FruHis group. Cigarette smoking can increase oxidative stress and inflammation and attenuate the beneficial effects of lycopene (44). In addition, a significant association between hypertension and IGF-1 levels was reported in previous studies (45). Therefore, our findings on the higher beneficial effects of lycopene plus FruHis intake, compared with the intake of lycopene alone, should be considered with caution. In addition, patients in the lycopene plus FruHis group had a higher prevalence of diabetes compared with other intervention groups. There is evidence indicating significant associations of diabetes with serum levels of PSA and the risk of PC (46, 47). The high prevalence of diabetes in the combined intervention may attenuate the reducing effects of lycopene plus FruHis intake on TPSA levels and this may explain why the TPSA-reducing effect of the combined treatment was not significant compared with the placebo. Further studies are required to confirm our findings.

### Strengths and limitations

This study had some strengths. In this study, we performed a comprehensive assessment (laboratory parameters, anthropometric measures, disease symptoms, and QoL) of patients with BPH before and after the trial. Participants in the four intervention groups were matched in terms of age and BMI and they were randomly allocated to the groups. Moreover, adherence to the interventions was high in our study, against the limitation by COVID-19 pandemic. Some limitations of our study should be taken into account. The most important limitation of this study is the low sample size. The main reason for the low sample size was limited funding as well as patient accessibility limitations due to the COVID-19 pandemic which made us unable to include a greater sample size. However, the number of patients included in the current study was reasonable for a pilot study. It should be noted that four patients were excluded during the trial and this dropout may affect the power of our study to detect significant effects. Also, the dropout among the intervention groups was different so the lycopene plus FruHis group contained 11 patients compared with the placebo group with 13 patients. In addition, due to limited funding, we could not assess tissue biomarkers of lycopene and FruHis to evaluate the influence of supplementation on tissue circulation of these compounds. In the current study, we prescribed lycopene and FruHis supplement as the two constituents of tomato products. It must be kept in mind that tomato products might have other effective constituents that influence BPH outcomes. Therefore, the effects of cooked tomato consumption, as a dietary intake on BPH outcomes might be different compared to the supplementation of lycopene and FruHis.

## Conclusion

FruHis intake strengthens the reducing effects of lycopene on IGF-1, TPSA, symptom score, BMI, and WC among patients with BPH. Except for IGF-1, these reductions were not statistically significant compared with the placebo, and the intakes of lycopene and FruHis alone, however, were clinically important. Such findings were not seen for FPSA and FPSA/TPSA ratios. Further clinical trials are needed to assess the effects of different dosages of lycopene and FruHis on BPH outcomes. In addition, future studies should examine the influence of other constituents in tomato/its products on BPH outcomes.

## Data availability statement

The raw data supporting the conclusions of this article will be made available by the authors, without undue reservation.

## Ethics statement

The studies involving human participants were reviewed and approved by the Tehran University of Medical Sciences (IR.TUMS.VCR.REC.1397.951). The patients/participants provided their written informed consent to participate in this study.

## Author contributions

AlS, AhS, MN, and MS designed the study. AlS, AmF, and EA collected the data. MY analyzed the data. AlS, AhS, and AlF drafted the manuscript. LJ-B and MK synthesized FruHis. All authors provided critical input and approved the final version of the manuscript.

## Footnotes

1
www.irct.ir
