# Supplementary material for: FruHis significantly increases the anti-benign prostatic hyperplasia effect of lycopene: A double-blinded randomized controlled clinical trial
Source: Front Nutr. 2022 Nov 3;9:1011836. doi: 10.3389/fnut.2022.1011836 (PMC9668902; doi:10.3389/fnut.2022.1011836)
Supplement: Supplementary file 1 [file Table_1.docx]

**Supplementary file**

**A)** Food record form used for the dietary assessment during the study

| **Days** | **Meals** | **Time** | **Food items** | **Serving** | **Amount (gr)** |
| --- | --- | --- | --- | --- | --- |
| Day 1 (working day) |  |  |  |  |  |
|  |  |  |  |  |  |
|  |  |  |  |  |  |
|  |  |  |  |  |  |
|  |  |  |  |  |  |
|  |  |  |  |  |  |
|  |  |  |  |  |  |
|  |  |  |  |  |  |
| Day 2 (working day) |  |  |  |  |  |
|  |  |  |  |  |  |
|  |  |  |  |  |  |
|  |  |  |  |  |  |
|  |  |  |  |  |  |
|  |  |  |  |  |  |
|  |  |  |  |  |  |
|  |  |  |  |  |  |
| Day 3 (weekend) |  |  |  |  |  |
|  |  |  |  |  |  |
|  |  |  |  |  |  |
|  |  |  |  |  |  |
|  |  |  |  |  |  |
|  |  |  |  |  |  |
|  |  |  |  |  |  |
|  |  |  |  |  |  |

**B) Physical activity record** used for the physical activity assessment during the study

| **Days** | **Activity description** | **Time** | **Duration** | **Intensity**  **(light, moderate, vigorous)** |
| --- | --- | --- | --- | --- |
| Day 1 (working day) |  |  |  |  |
|  |  |  |  |  |
|  |  |  |  |  |
|  |  |  |  |  |
|  |  |  |  |  |
|  |  |  |  |  |
|  |  |  |  |  |
|  |  |  |  |  |
| Day 2 (weekend) |  |  |  |  |
|  |  |  |  |  |
|  |  |  |  |  |
|  |  |  |  |  |
|  |  |  |  |  |
|  |  |  |  |  |
|  |  |  |  |  |
|  |  |  |  |  |
